# Non-enzymatic sensor for determination of glucose based on PtNi nanoparticles decorated graphene

**DOI:** 10.1038/s41598-020-73567-2

**Published:** 2020-10-08

**Authors:** Risheng Li, Xu Deng, Longfei Xia

**Affiliations:** 1 Shaanxi Provincial Land Engineering Construction Group Co., Ltd.; Institute of Land Engineering and Technology, Shaanxi Provincial Land Engineering Construction Group Co., Ltd.; Key Laboratory of Degraded and Unused Land Consolidation Engineering, the Ministry of Natural Resources, Xi’an, 710075 China; 2 Key Laboratory of Analytical Chemistry for Life Science of Shaanxi Province and Key Laboratory of Medicinal Resources and Natural Pharmaceutical Chemistry of the Ministry of Education, Shaanxi Normal University, Xi’an, 710062 China

**Keywords:** Analytical chemistry, Nanoscale materials

## Abstract

Diabetes has become a universal epidemic in recent years. Herein, the monitoring of glucose in blood is of importance in clinical applications. In this work, PtNi alloy nanoparticles homogeneously dispersed on graphene (PtNi alloy-graphene) was synthesized as a highly effective electrode material for glucose detection. Based on the modified PtNi alloy-graphene/glass carbon (PtNi alloy-graphene/GC) electrode, it is found that the PtNi alloy-graphene/GC electrode exhibited excellent electrocatalytic performance on glucose oxidation. Furthermore, the results from amperometric current–time curve show a good linear range of 0.5–15 mM with the limit of detection of 16 uM (S/N = 3) and a high sensitivity of 24.03 uAmM^−1^ cm^−2^. On account of the good selectivity and durability, the modified electrode was successfully applied on glucose detection in blood serum samples.

## Introduction

Glucose sensors have attracted more attention in the medical application of blood glucose sensing. Especially for diabetic patients, the precise monitoring and careful control of the level of glucose in human blood are essential^1,2^. For nowadays, most of the traditional methods for measuring blood glucose levels are involved in electrochemical or colorimetric readout systems. These tests are mainly based on enzymatic methods owning to the advantage of simple, high sensitivity, rapid and low-cost^3–6^. However, the enzymatic glucose sensors, usually modified based on glucose dehydrogenase (GDH) or glucose oxidase (GOx), which are instability, easily affected^5,6^. Thus, it is worthwhile to develop nonenzymatic biosensors which allow glucose to be oxidized directly on the electrode surface to overcome these disadvantages.

Graphene and its hybrids, owing to its high surface area, good electrical conductivity and outstanding chemical stability, are wildly utilized in many electrocatalytic application fields^7–10^. They are also good candidates for developing nonenzymatic sensors^11–14^. In recent years, metals and their compounds were explored for application in nonenzymatic biosensors with enhanced properties^15–17^. In these electrodes, platinum was the most promising candidate for glucose oxidation. However, pure Pt is expensive and easily contaminated by chemisorbed intermediates of glucose oxidation^18,19^. To overcome these obstacles, Pt-based alloys decorated on graphene as glucose sensors have been studied in recent years^20,21^. The introduce of other mental elements, such as Co, Pd and Ni, into the bimetallic systems could change the local strain and the effective atomic coordination number of Pt on surface^22–24^. Thus, these Pt-based alloy catalysts could effectively offer electrodes with desirable catalytic efficiency for glucose oxidation compared to pure Pt catalysts.

Herein, PtNi alloy nanoparticles were synthesized on graphene as a highly effective electrode material for the detection of glucose in this work. Graphene is chosen as the supporting substrates, as the highly conductive graphene nanosheets could give rise to the PtNi catalysts with larger active surface areas and also improve the electron transport during the glucose oxidation reaction^25^. In this work, platinum was reduced by substitution reaction to form PtNi alloy with majority of Pt on surface to reduce catalyst cost. On the other hand, nickel and nickeloxides/hydroxides are not only promoters but could also be catalysts for the relevance reaction of glucose sensing^26,27^ After the PtNi alloy-graphene catalysts were decorated on glassy carbon(GC) electrode, the linearity, limit of detection (LOD) and sensitivity of the PtNi alloy-graphene/GC electrode was explored to evaluate the electrocatalytic activities for glucose oxidation. The results found that highly selective nonenzymatic glucose detection were obtained with the PtNi alloy-graphene/GC electrode compared to Pt/C/GC electrode and other previous reported non-enzymatic glucose sensors. Moreover, the modified electrode also showed exceptional stability and reproducibility towards glucose. Finally, the modified electrode effectively applied to the determination of glucose in the blood serum samples.

## Experimental

### Synthesis of graphene decorated PtNi alloy nanoparticles

The Ni nanoparticles were firstly synthesized on graphene via hydrothermal method. 10 mg graphene was added into the solution of 200 mg Ni(CH_3_COO)_2_·4H_2_O, 400 mg NaH_2_PO_2_·H_2_O and 10 mg PVP dissolved in 10 ml distilled water. After that, the mixture was kept in oven at 120 °C for 12 h. After filtered and dried, the Ni-graphene was added into the aqueous H_2_PtCl_6_·6H_2_O solution (40 mL, 2 mM) at room temperature for 12 h to synthesis PtNi alloy-graphene by chemical reduction reaction.

### Preparation of the modified glassy carbon electrode

The glassy carbon (GC) electrode was firstly polished with alumina slurries to mirror smoothness and then rinsed with ethanol and distilled water respectively. After dried at room temperature, 2 mg of PtNi alloy-graphene was dispersed in 5 mL dimethylformamide (DMF) under ultrasonic agitation to prepare as the catalyst ink. After that, 10 μL of PtNi alloy-graphene suspension was dropped onto the surface of GC to form PtNi alloy-graphene/GC electrode. The resulting electrode was finally covered with 5 μL of Nafion solution to make the catalysts stable. As a comparison, the commercial Pt/C catalyst (20 wt% Pt supported on carbon) modified GC electrode was also prepared via the same method.

### Apparatus and measurements

X-ray diffraction (XRD) was used to test the crystalline structures of the catalysts by using Cu Ka (1.54 Å) radiation. The surface characteristics of the catalysts were investigated by X-ray photoelectron spectroscopy (XPS) with monochromatic Al Ka line at 1486.6 eV. The binding energies were calibrated with respect to the C 1s peak at 284.6 eV. The morphology was confirmed by transmission electron microscopy (TEM, JEOL-2011) with an accelerating voltage of 120 kV. All the electrochemical experiments were carried on a electrochemical workstation (CHI 760E) at room temperature with a conventional three-electrode system by using an Ag/AgCl as the reference electrode and a platinum mesh as counter electrode respectively. The glucose detection was conducted in the solution of β-d-glucose (Aldrich-Sigma) in phosphate buffer solution (PBS, 0.1 M, pH7.4).

## Results and discussion

### Characterization of PtNi alloy-graphene

**Figure 1:**
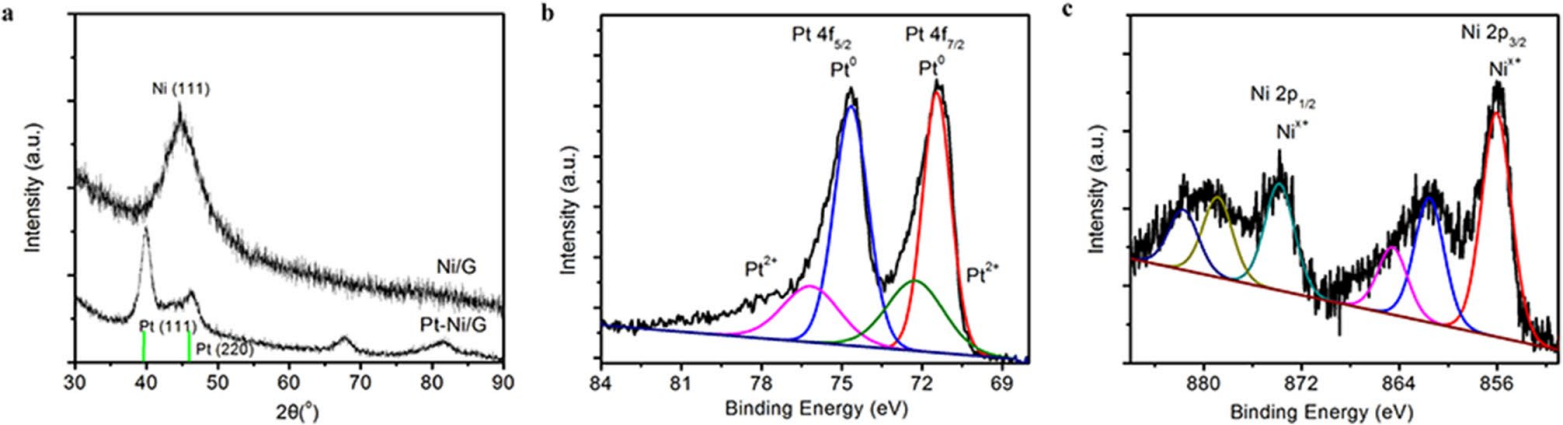
(**a**) X-ray diffraction ptterns of Ni-graphene and PtNi alloy-graphene, XPS spectra of (**b**) Pt 4f and (**c**) Ni 2p for PtNi alloy-graphene.

The X-ray diffraction patterns of Ni-graphene and PtNi alloy-graphene are shown in Fig. 1a. For Ni-graphene nanoparticles, the peak at 44.62° is indexed to the face-centered cubic (fcc) Ni (111). After the chemical reduction reaction, the Ni peak is vanished. And the broad peaks of PtNi alloy nanoparticles appeared with higher 2θ values compared to that of Pt (111) and (200) (JCPDS 04–0802), suggesting the formation of PtNi alloys^23,26^. The chemical state of PtNi alloy-graphene nanoparticles was examined by XPS as shown in Fig. 1b. The peaks of Pt 4f at 71.38 and 74.57 eV present metallic Pt, and the oxidized state of Pt also appears at 72.28 and 76.18 eV. The XPS spectra of Ni 2p in Fig. 1c show that Ni is in oxidized state, corresponding to NiO, Ni(OH)_2_, and NiOOH, respectively. The major component of the PtNi alloy-graphene catalyst is nickel hydroxide Ni(OH)_2_^23^.

**Figure 2:**
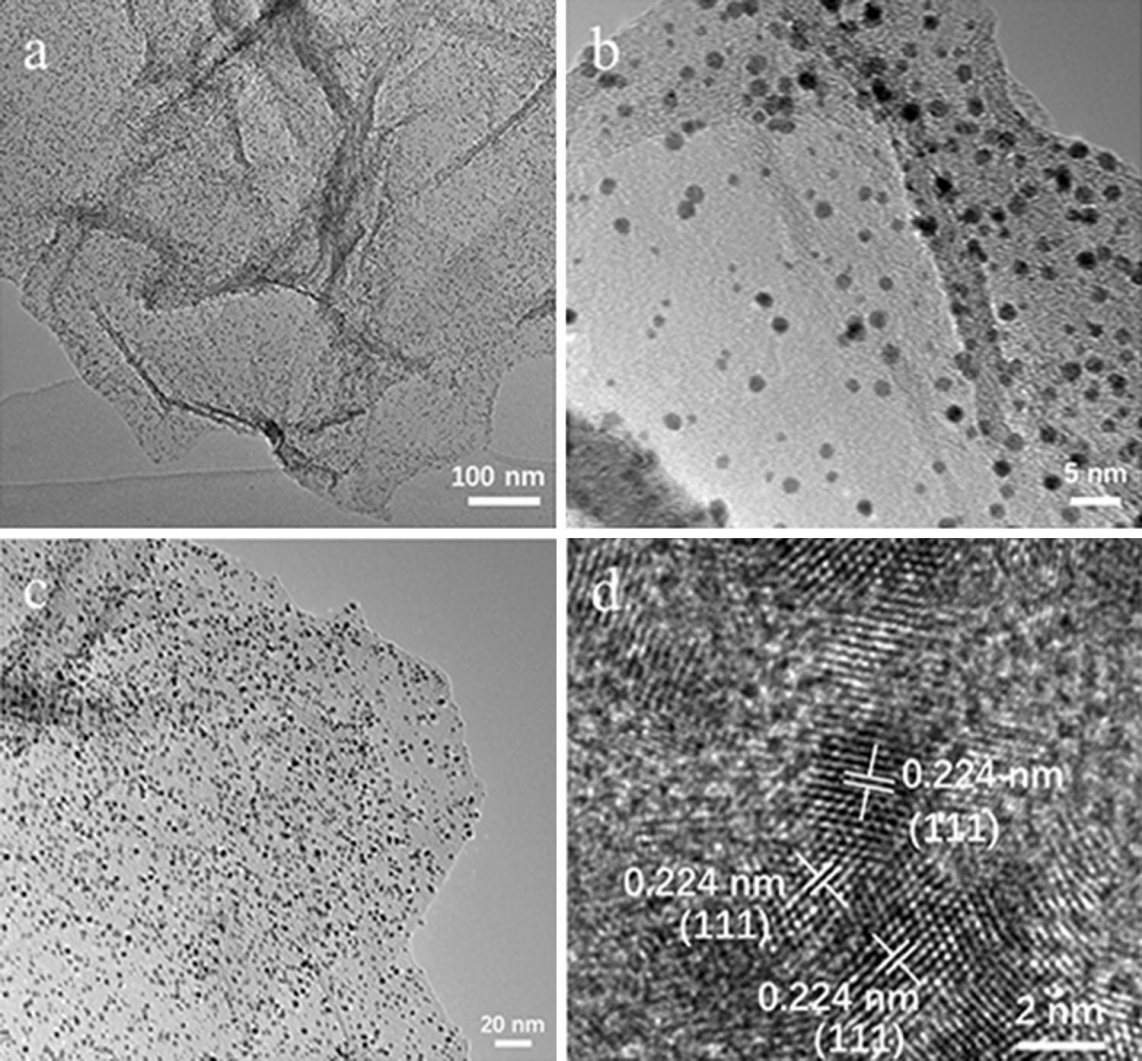
TEM image (**a**,**b**) of Ni-graphene. (**c**) TEM and (**d**) HRTEM images of PtNi alloy-graphene.

The surface morphologies of Ni-graphene and PtNi alloy nanoparticles were examined by TEM. As shown in Fig. 2a, nickel nanoparticles are homogeneous distributed on graphene. The size of the nickel nanoparticles is around 2–3 nm (Fig. 2b). After the chemical reduction reaction, the PtNi alloy nanoparticles maintain the homogeneous dispersion with particle size of around 2–3 nm (Fig. 2c). Figure 2d presents a lattice resolved high resolution TEM (HRTEM) image of PtNi alloy nanoparticles. The lattice fringes with an interlayer spacing of 2.24 Å is consistent with the (111) crystallographic planes of face-centered cubic (fcc) PtNi alloys^26–28^.

### Electrocatalytic activity on glucose oxidation

**Figure 3:**
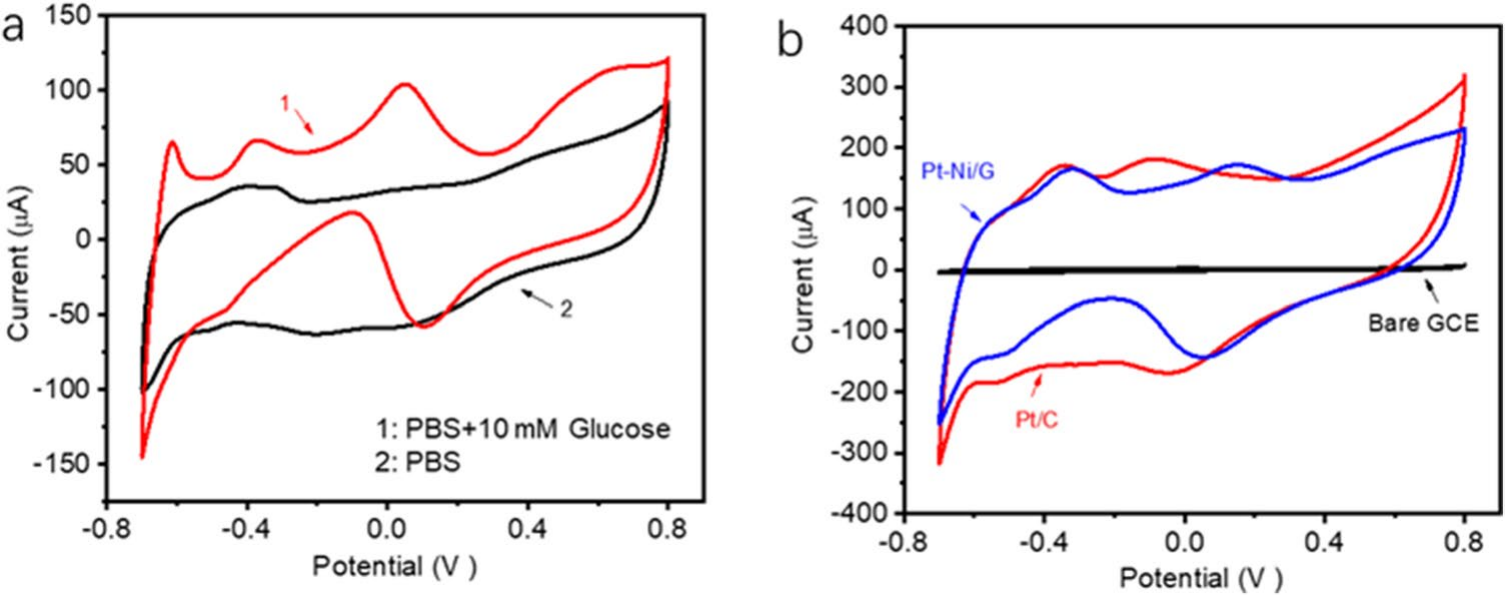
(**a**) CVs of PtNi alloy-graphene/GC electrode in the absence and present of 10 mM glucose in 0.1 M PBS solution at scanning rate of 10 mV/s; (**b**) CVs of PtNi alloy-graphene/GC, commercial Pt/C/GC and bare GC electrodes in 0.1 M PBS with 10 mM glucose at scanning rate of 30 mV/s.

Cycle voltammetries (CVs) of PtNi alloy-graphene/GC electrode with and without 10 mM glucose in 0.1 M PBS solution at scanning rate of 10 mV/s is shown in Fig. 3a. In the blank PBS solution, the peaks at potential of − 0.65 to − 0.23 V are attributed to hydrogen desorption in positive scan and hydrogen adsorption process in negative scan respectively. However, in the presence of 10 mM glucose, more oxidation peaks are observed on PtNi alloy-graphene/GC electrode. The peak at around -0.30 V is ascribed to the formation of adsorbed intermediates on PtNi alloy nanoparticles due to the electrochemical adsorption of glucose. Furthermore, the adsorbed glucose intermediates are oxidized as the potential scanning to higher positive value, resulting in the current peak at around 0.10 V. However, the formation of metal oxides on surface would further inhibit the direct oxidation of glucose at higher potential beyond 0.4 V. But the reduction of metal oxides would be reduced in the process of negative scan. With the reduction of metal oxides in the process of negative scan, more active sites on surface would expose for glucose oxidation process, contributing to the large oxidation peak at around 0.1 V.

CVs of PtNi alloy-graphene/GC, commercial Pt/C/GC and bare GC electrodes were tested in 0.1 M PBS containing 10 mM glucose at scanning rate of 30 mV/s (Fig. 3b). It is obvious that there is no catalytic performance on glucose oxidation by GC electrode. As expected, the PtNi alloy-graphene/GC electrode exhibits obvious catalytic activities for glucose oxidation comparing to that of Pt/C/GC electrode. For non-enzymatic glucose sensors, the atoms on electrode surface directly work with glucose molecules. The oxidation process starts from the adsorption of glucose molecules on metal surface. Based on the generated bond between glucose and metal during adsorption step, the metal catalysts will promote the oxidation of glucose. Finally, the gluconate or other intermediates depart from the metal surface owing to the lowered glucose-metal bond. Therefore, a moderate bond strength is necessary for an effective glucose oxidation process. It is known that alloying with transition metals, the electronic properties of Pt atoms on surface could be changed, that is directly associated to the adsorption strengths for the glucose intermediate species^29,30^. In this work, the modified electronic properties of Pt by alloying with Ni can weaken the adsorption strength of gluconate or intermediates species. As the coverage of glucose intermediates on electrode surface decreases, the electrode could promote more active surface areas for glucose oxidation, which contribute to the enhanced electrocatalytic activity towards glucose oxidation.

**Figure 4:**
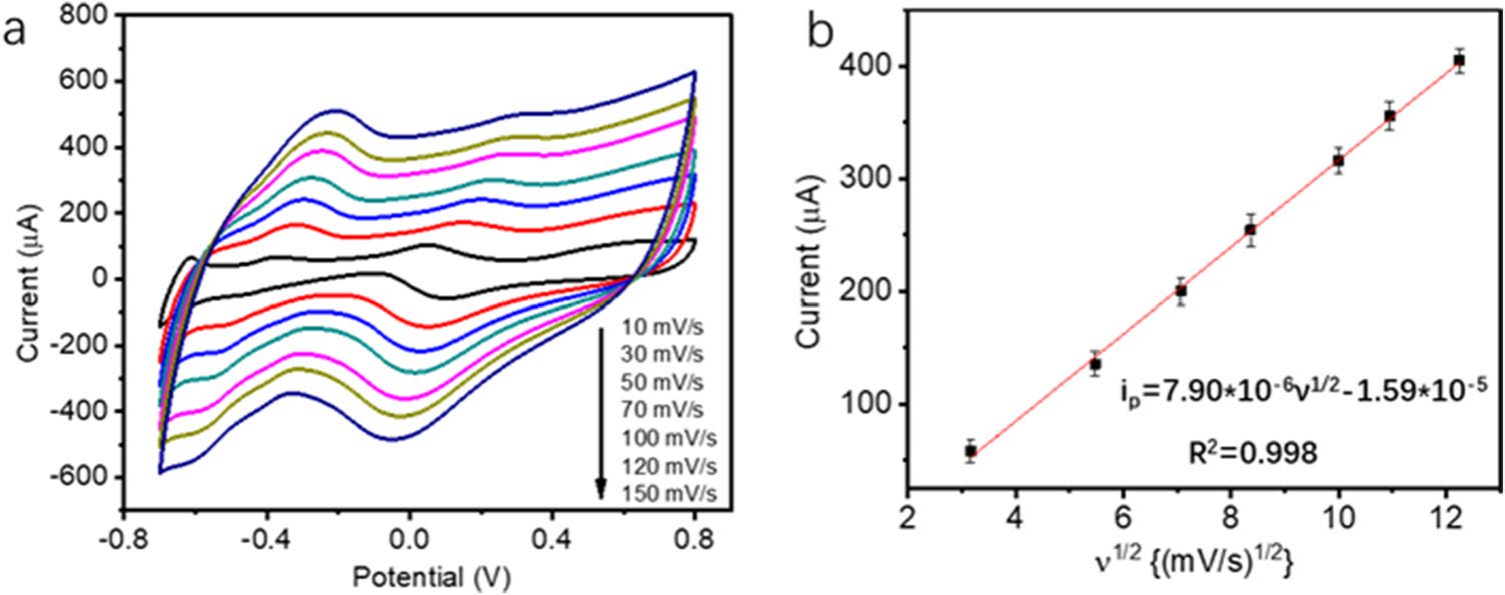
(**a**) CVs of PtNi alloy-graphene/GC electrode in 0.1 M PBS with 10 mM glucose at various scan rate from 10 to 150 mV/s. (**b**) Corresponding plot of peak current at 0.1 V vs. the square root of the scan rate.

To further understand the reaction mechanism, the voltammetric response of glucose oxidation on PtNi alloy-graphene/GC electrode with various scan rate was studied. Figure 4a presents the CVs of PtNi alloy-graphene/GC electrode at scanning rates from 10 to 150 mV s^−1^ in 0.1 M PBS containing 10 mM glucose. It is notable that the peak currents gradually increase with the increasing scanning rates and the potential peaks also shift slightly. Correspondingly, the peak current at 0.1 V versus the square root of the scan rate was calculated. As shown in Fig. 4b, the peak current is linearly proportional to the square root of the scan rate. The results indicate that the electrochemical oxidation of glucose on PtNi alloy-graphene/GC electrode is a diffusion-controlled reaction process with a fast reaction kinetics for glucose oxidation.

### Non-enzymatic sensing of glucose

**Figure 5:**
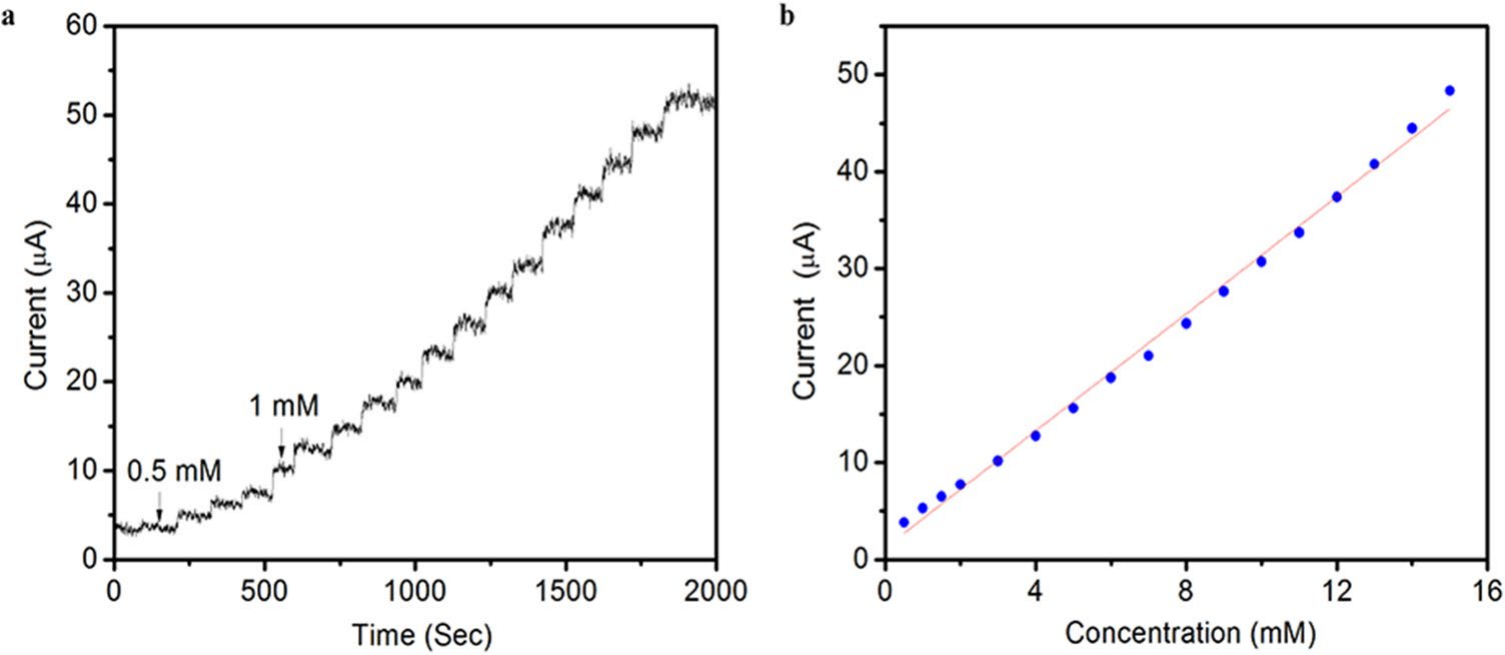
(**a**) Amperometric response of the PtNi alloy-graphene/GC electrode to successive addition of 0.5 and 1 mM glucose in 0.1 MPBS at an applied potential of 0.3 V and (**b**) corresponding calibration curve.

As an enzyme-free biosensor, it is necessary to explore the analysis of glucose oxidation on PtNi alloy-graphene catalysts by amperometric current–time tests. The electrochemical response for successive addition of glucose at potential of 0.3 V is exhibited in Fig. 5a. After the injection of glucose into solution, the current response increases to a steady state value immediately. The results suggest that the PtNi alloy-graphene/GC electrode have a quick response to the change of glucose concentration. Moreover, the response behavior of PtNi alloy-graphene/GC electrode is linearly in the range of 0.5 to 15 mM with a correlation coefficient of 0.9958 (Fig. 5b). The sensitivity is calculated to be about 24.03 uA/mM cm^2^ and the detection limit is estimated to be 16 uM with a signal/noise ratio (S/N) of 3. This result is comparable to those Pt and Pt alloy electrodes reported in other literatures as shown in Table 1.

**Table 1:** Comparison of different non-enzymatic Pt and Pt-base glucose sensors.

Electrode materials	Sensitivity (uAmM^−1^ cm^−2^)	Linear range (mM)	LOD (uM)	Working potential (V)	Reference
PtNi alloy-graphene/GC	24.03	0.5–15	16	+ 0.3	This work
Hollow Pt–Ni–graphene	30.3	0.5–20	2	− 0.35	^23^
Pt_3_Ni_7_/MWCNTs-Nafion	940	~ 15 mM	0.3	− 0.3	^26^
Ni@Pt/C	66.9	0.1–30.1	30.0	− 0.10	^29^
Pt/MWNTs/graphene	11.06	1.0–7.0	387.0	+ 0.40	^31^
Pt–Pd nanowire arrays	27.6	Up to 10	not give	+ 0.2	^32^
Pt_3_Ru_1_/GC	31.3	Up to 4	0.3	0.1	^33^
Au/MRGO/PtAuNFs/Ch-GOx/PDDA	17.85	0.01–8	1	0.6	^34^

### Interference of the sensor

**Figure 6:**
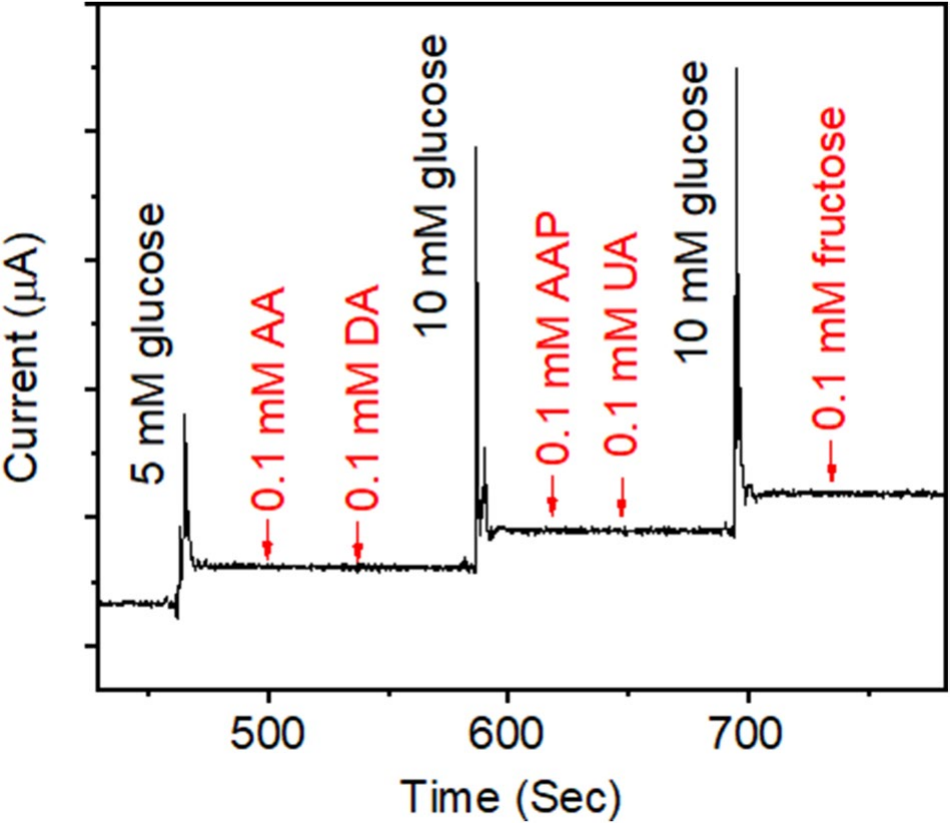
Amperometric response of the PtNi alloy-graphene/GC electrode at 0.3 V to the addition of AA, DA, AAP, UA, fructose and glucose.

For sensing applications, the oxidation current of interfering reagents is necessary to investigate for sensors. Figure 6 shows the amperometric current responses to AA, DA, AAP, UA, frucose and glucose in 0.1 M PBS at an applied potential of − 0.1 V. The as-prepared electrode has exact response from the direct glucose oxidation with 5 and 10 mM. Furthermore, it presents a good capability of anti-interference as the oxidation current of interfering reagents is invisible to observe. The good selectivity of PtNi alloy-graphene/GC electrode implies its practical application for glucose detection.

### Stability and reproducibility of the sensor

**Figure 7:**
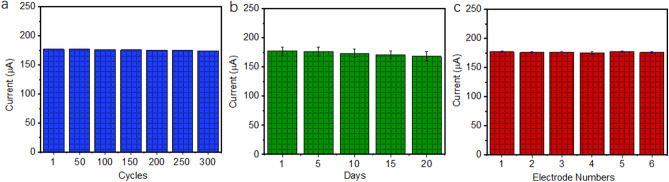
The stability of the PtNi alloy-graphene/GC electrode for glucose detection (**a**) at different cycles and (**b**) for days. (**c**) The current responses of six PtNi alloy-graphene/GC electrodes with 10 mM glucose.

The stability and reproducibility of the as-prepared electrode were also evaluated. The CVs of PtNi alloy-graphene/GC electrode in 0.1 M PBS with 10 mM glucose at scan rate of 30 mV/s were tested for 300 successive cycles. The peak currents of the 1st, 50th, 100th, 150th, 200th, 250th and 300th cycles at around 0.1 V were used to depict the stability of the electrode. As shown in Fig. 7a, after 300 successive cycles, the peak current intensity maintains 98.45% of the initial value. The durability of PtNi alloy-graphene/GC electrode was also investigated by recording its peak current response to 10.0 mM glucose during stored in a refrigerator at 5 °C for 20 days. It has been seen in Fig. 7b, the as-prepared electrode could steadily retain 95.2% of its initial sensitivity after 20 days. The above results indicate an extraordinary stability of the modified electrode.

The peak current response of six electrodes modified identically to 10 mM glucose at around 0.1 V were tested for the reproducibility. As shown in Fig. 7c, the PtNi alloy-graphene/GC electrode displays a relative standard deviation (RSD) around 1.5%, suggesting that the sensor is highly reproducible.

### Practical applicability of the sensor

Practical applicability of the PtNi alloy-graphene/GC electrode on human serum samples was applied to verify the reliability and feasibility of the sensor. The human blood serum samples were acquired from Shaanxi people’s Hospital, China. And the detection of the glucose concentration was conducted by standard method in hospital. The results of glucose determination in human serum samples is presented in Table 2. The recoveries are ranging from 98.6 to 103.3%. The good recoveries indicate that the PtNi alloy-graphene could establish as an effective electrode for measuring glucose in real samples.

**Table 2:** Determination of glucose in human serum samples by PtNi alloy-graphene/GC electrode.

Added (mM)	Found (mM)	Recovery (%)	RSD (%)^a^
3.0	3.10	103.3	2.01
6.0	5.92	98.6	2.32
9.0	8.90	98.9	2.25

^a^The Related standard deviation (RSD) is calculated according to n = 3.

## Conclusion

In summary, we developed a facile method to synthesis PtNi alloy nanoparticles decorated on graphene for glucose detection.. Cycle voltammetry results suggested that the PtNi alloy-graphene/GC electrode has an enhanced performance towards glucose oxidation in comparison with Pt/C electrode. The amperometric response of the PtNi alloy-graphene/GC electrode exhibited a good linear range of 0.5–15 mM with the limit of detection of 16 mM (S/N = 3) and a high sensitivity of 24.03 uAmM^−1^ cm^−2^. The modified electrode also presented a higher selectivity, good stability and reproducibility. These results suggest that the PtNi alloy-graphene/GC electrode have the potential of utilizing as a novel candidate for nonenzymatic glucose sensors.
